# The future excess fraction model for calculating burden of disease

**DOI:** 10.1186/s12889-016-3066-1

**Published:** 2016-05-11

**Authors:** Lin Fritschi, Jayzii Chan, Sally J. Hutchings, Tim R. Driscoll, Adrian Y. W. Wong, Renee N. Carey

**Affiliations:** School of Public Health, Curtin University, Perth, Australia; Department of Mathematics and Statistics, Curtin University, Perth, Australia; Department of Epidemiology and Biostatistics, Imperial College London, London, UK; School of Public Health, University of Sydney, Sydney, Australia

**Keywords:** Burden of disease, Methodology, Policy, Prevention

## Abstract

**Background:**

Estimates of the burden of disease caused by a particular agent are used to assist in making policy and prioritizing actions. Most estimations have employed the attributable fraction approach, which estimates the proportion of disease cases or deaths in a specific year which are attributable to past exposure to a particular agent. While this approach has proven extremely useful in quantifying health effects, it requires historical data on exposures which are not always available.

**Methods:**

We present an alternative method, the future excess fraction method, which is based on the lifetime risk approach, and which requires current rather than historical exposure data. This method estimates the future number of exposure-related disease cases or deaths occurring in the subgroup of the population who were exposed to the particular agent in a specific year. We explain this method and use publically-available data on current asbestos exposure and mesothelioma incidence to demonstrate the use of the method.

**Conclusions:**

Our approach to modelling burden of disease is useful when there are no historical measures of exposure and where future disease rates can be projected on person years at risk.

## Background

Burden of disease estimates are used widely to assist in policy-making and prioritizing of actions [1]. The most commonly used method for estimating burden of disease is the attributable fraction approach. Using this approach, estimates of the proportion of disease cases or deaths in a specific year are calculated as being attributable to past exposure [2]. Most of these type of studies obtain the proportion of the population exposed from historical surveys representative of the population for which the burden is being calculated [3]. While this approach has proven extremely useful in quantifying health effects to guide prevention methods [4], it requires that data on historical exposures are available.

**Fig. 1:**
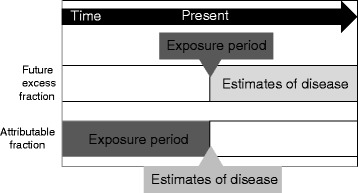
Comparison between attributable fraction approach and future excess fraction approach

We have developed an alternative method, based on the lifetime risk approach, which estimates the excess exposure-related disease cases or deaths occurring in the future in the subgroup of the population who were exposed to the agent in a specific year [5]. This method, termed the future excess fraction (FEF) method can be conceptualized as the creation of a virtual cohort, with some cohort members exposed to a particular agent in a specific year and others not. This cohort is followed through time into the future, and the number of cases of disease in the whole cohort is estimated (Fig. 1). Then, using the prevalence of the exposure and the relative risk of disease due to that exposure, we can calculate the number of exposure-related disease cases or deaths occurring over a specified future period in the subgroup of the population who were exposed to the particular agent in a specific year due to their exposure.

The FEF approach can be used when no historical exposure data are present and projections of the disease can be made on future person years at tirk. The primary value of the method is that it allows us to look at hypothesized future scenarios in which nothing changes and, in comparison, what would happen with various planned interventions.

In this paper we explain the FEF method and demonstrate its use with data on occupational asbestos exposure and mesothelioma.

### The lifetime risk method

The risk of developing cancer or other conditions is often presented as the probability of developing the disease over a lifetime or the ‘lifetime risk’. For example, in 2011, the risk for Australian males of being diagnosed with mesothelioma before their 85th birthday was 1 in 130 or 0.8 % [6]. The ‘lifetime’ we refer to here is the lifetime of all members of a cohort, as a specific individual's risk of cancer varies depending on their own risk factors [7].

There are several ways to calculate lifetime risk. The Cumulative Risk is the probability of developing or dying from a disease up to a specific age. It is calculated by summing the age-specific incidence rates for each year up to an upper age limit and is usually expressed as a 1 in *n* chance [8]. It may be understood as a standardized rate which has used 1.0 for each age weighting factor. The Cumulative Risk does not take into account the competing mortality risk and is highly dependent on the upper age limit chosen [7]. Therefore a cumulative risk is only valid if an individual can only have a maximum of one disease event. It provides an approximation of the lifetime risk of disease when the upper age limit is close to the population's average life expectancy but typically this is an over-estimate of the true lifetime risk.

A more realistic estimate of the lifetime risk was developed by Goldberg et al. [9] who incorporated the competing risk of mortality through the use of life tables and a hypothetical birth cohort to calculate the number of cases that would occur within each age band. Further work by Sasieni et al. [10] included adjustment for multiple disease events.

Bender et al. [7] proposed that the expected numbers of cases of diseases should be calculated from the disease-free population's person-years rather than the total population. In their revised Person-Years (PY) model, the lifetime risk is calculated as the total number of cancers experienced by the cohort from age x divided by the number of cohort survivors at age x.

We based our FEF model on the PY model [7] with some changes (described below) to make it more appropriate for our requirements.

## Methods

We use the following naming conventions:

*N*_*P(t)*_ is the number of individuals in population *P* at time *t*

*N*_*e(t= 0)*_ is the number of individuals in population *P* who are exposed to the agent in the index year

*D*_*P(t)*_ is the number of disease cases in population *P* at time *t*

*LR*_*P*_ is the lifetime risk of disease in population *P*

*FN*_*P*_ is the total number of disease cases in population *P* over a lifetime

The key steps of our FEF approach are outlined below.

Step 1. Define the index year (t = 0) as the year in which the study population is defined and the exposure categorization is made. Define the population of interest, which may be the current resident population perhaps limited by age, sex or other factors. For simplicity, in the methodological overview we have stratified by age but not sex. The total number of individuals in the population in the index year is denoted by *N*_*p(t = 0)*._
Step 2. Estimate the number of individuals in the population of interest who are exposed to the agent in the index year (*N*_*e(t = 0)*_). Note that there may be a range of exposures in this group (in both level and duration up to t = 0). Also note that those who have been exposed in the past to the agent but are not exposed in the index year will be classified as unexposed. The number of unexposed individuals is *N*_*u(t = 0)*_.
Step 3. Estimate the future Person Years at Risk (PYAR) for the population of interest irrespective of exposure using standard life tables. These tables use national death rates to estimate the probability that an individual of a certain age is alive at each age in the future. Single decrement tables assume that an individual continues to contribute person years to the cohort until they die. Double decrement tables, which take into account the competing probabilities of death from any cause as well as incidence of the disease of interest can be used if the disease of interest is an absorbing state with no possibility of a return transition. Multi-state life tables provide for the possibility of return transitions but increase further the data requirements and complexity of the method.
Step 4. Estimate the age-specific death or incidence rates for the disease of interest in each age group at the midpoint in the index year.
Step 5. Calculate the projected future age-specific rates of disease for each future period of, for example, 10 years (which we will refer to as the estimation interval). This estimate is irrespective of exposure*.* Projection of future rates usually requires an estimate of historical rates and a projection model and methods will differ according to the disease of interest.
Step 6. Calculate the estimated number of disease cases (*D*_*t*_) at each estimation interval by multiplying the age-specific rate at the midpoint of the estimating interval by the person-years in the relevant cell. Sum these to arrive at the total number of disease cases in the lifetime of the population (*FN*_*p*_).
Step 7. Estimate the lifetime risk in the population (*LR*_*p*_) for the disease. This estimate is irrespective of exposure and calculated per individual aged *i* for each estimation interval *j* according to the equation:
  $$ L{R}_P = {\displaystyle \sum_{i=l}^{i=u}}\frac{{\displaystyle {\sum}_{j=l}^{j=u}}{D}_{ij}}{N_{p\left(t=0\right)}} $$
  Where *u* = upper limit and *l* = lower limit of both age and year
Step 8. Obtain an effect measure such as a relative risk estimate (*RR*) for each disease-agent pairing from existing literature. This should be an effect measure which corresponds with the definition of exposure used in step 2. It is possible to use multiple effect measures corresponding to multiple levels of exposure, but for simplicity we have assumed there is just one level of exposure.
Step 9. Calculate the lifetime excess risk (*LR*_*x*_) for an individual exposed to the agent in the index year. Theoretically, this should be calculated as:
  $$ L{R}_x = L{R}_u*\left(RR\hbox{--}\ 1\right) $$
  where (*RR* - 1) is the excess risk in the exposed relative to the unexposed and *LR*_*u*_ is the baseline risk in the population in the absence of exposure. This assumes that the ratio between the excess risk of disease in the exposed and the risk in the unexposed at baseline equals the ratio of the lifetime risks, as *LR*_*x*_*/LR*_*u*_ *= (R*_*e*_*– R*_*u*_*)/R*_*u*_ *= R*_*x*_*/R*_*u*_*,* where *RR* = *R*_*e*_*/R*_*u*_, *R*_*e*_ is the risk of disease in the exposed, *R*_*u*_ is the risk in the unexposed and *R*_*x*_ is the excess risk due to the exposure [2]. This is similar to an assumption made in the attributable fraction approach. However, *LR*_*u*_ is not usually available so *LR*_*p*_ (lifetime risk in the whole population) is sometimes used. Because *LR*_*p*_ is calculated for the whole population (including both exposed and unexposed individuals) it is higher than the baseline risk in the absence of exposure (*LR*_*u*_). For agent-disease combinations with low prevalence and/or low relative risk estimates *LR*_*u*_ is very similar to *LR*_*p*_. However the difference can be significant for agent-disease combinations with high prevalence and/or high relative risks. We have therefore modified this equation (see Appendix) to arrive at the following:
  $$ L{R}_x=\frac{\left(L{R}_P*{N}_{p\left(t=0\right)}\right)\left(RR-1\right)}{N_{u\left(t=0\right)}+{N}_{e\left(t=0\right)}*RR} $$
  This equation only holds under the assumption that *RR* is greater than 1.0.
Step 10. Estimate the Future Excess Number (FEN) of disease cases related to exposure that can be expected to occur over the lifetime of the exposed subcohort in the index year by multiplying the individual lifetime excess risks (*LR*_*x*_) by an estimate of numbers exposed (*N*_*e(t = 0)*_). Although it is a similar concept we have not called this the Attributable Number to avoid confusion with the terminology used in the attributable fraction approach.
Step 11. Calculate the Future Excess Fraction (FEF) of diseases in the exposed population which are due to exposure in the index year by dividing the FEN by the total expected number of disease cases in the population. The total expected number of diseases in the population is equal to the product of the lifetime risk in the population multiplied by the number of individuals in the cohort (exposed and unexposed).
  $$ FEF=\frac{FEN}{L{R}_P*{N}_{p\left(t=0\right)}} $$

### Example data

Our example is that of the proportion of future mesotheliomas which are attributable to occupational exposure to asbestos in Australia in 2012.

1. The index year was 2012. The population of interest (the “virtual cohort”) was the 2012 working age population in Australia (18–65 years), divided into an initial two year age band (18–19 years) and subsequent five-year age bands, and stratified by sex.
2. The number of individuals who were occupationally exposed to asbestos in 2012 (*N*_*e(t = 2012)*_) was extrapolated from a national survey [11].
3. The Person-Years at Risk for the working age population was calculated using the 2012 mid-year population statistics [12] and a matrix of future individual person-years which was truncated according to a double decrement table (death and first diagnosis of mesothelioma as endpoints). Because of the poor prognosis of mesothelioma it can be thought of as an absorbing state.
4. Age- and sex-specific incidence rates for mesothelioma at the midpoint of 2012 were obtained from the national cancer registry [6].
5. Projected future rates of mesothelioma were calculated for each age group, sex and 5-year period using CanProj [13] for the period 2010 until 2094. CanProj is an R package which uses a decision tree to determine and conduct the most appropriate forward projection model, whether age-period-cohort (APC; “power5”) or a loglinear regression model (“hybrid”).
6. The age-specific future mesothelioma rates in each estimation interval were multiplied by the age-specific person-years at risk of the working age population in the same estimation interval and summed to estimate the total number of mesotheliomas in each estimation interval (*D*_*pt*_). The sum of all *D*_*pt*_ is the *FN*_*p*_*.*
7. The sex-specific lifetime risk of disease (*LR*_*p*_) was then calculated as *FN*_*p*_ divided by *N*_*p(t = 2012)*_*.*
8. Relative risk of mesothelioma for a member of the population occupationally exposed to asbestos was obtained from a Spanish study [14]. This study was chosen as it compared subjects who had never been exposed to asbestos at work with those exposed occupationally to asbestos in similar working circumstances as in Australia.

The formulae in Steps 9–11 were then used to calculate the excess lifetime risk (*LR*_*x*_) of mesothelioma due to occupational exposure in 2012 for the population aged 18–65 years in 2012, as well as the FEN and FEF which are the future excess number and fraction of mesotheliomas in the cohort from 2012 to 2094 which occur in those occupationally exposed to asbestos in 2012 and are attributed to occupational asbestos exposure.

For comparison purposes we undertook an analysis using the attributable fraction (AF) method. We used the same index year, 2012 working population, proportion exposed in 2012, and relative risk as in the example above (steps 1, 2, and 8). We predicted the AF of mesotheliomas using the formula:

$$ \mathrm{A}\mathrm{F} = \left[ \Pr \left({\mathrm{E}}_{2012}\right)\ *\ \left(\mathrm{R}\mathrm{R}-1\right)\right]\ /\ \left[1 + \Pr \left({\mathrm{E}}_{2012}\right)\ *\ \left(\mathrm{R}\mathrm{R}-1\right)\right] $$

We used the same method as above (steps 3 to 6) to calculate the number of mesotheliomas occurring only in the year 2052. The attributable number was equal to the AF multiplied by the number of mesotheliomas in 2052.

## Results

Table 1 summarizes the results for the lifetime risk model. Lifetime risk of mesothelioma in the 2012 working age population was estimated at 33/10 000 for males and 15/10 000 for females. Of future mesotheliomas in the current working age population about 32 % in males and <1 % in females are likely to be due to occupational exposure in those workers exposed to asbestos in 2012 (FEF).

**Table 1:** Results from the future excess fraction method estimating the lifetime risk of mesothelioma (2012 to 2094) in the Australian working population exposed to asbestos in the year 2012

	Males	Females
Proportion of working age population exposed to asbestos in 2012 [11]	0.0347	0.0004
Number of working age people in 2012 exposed to asbestos	253 110	2757
Lifetime risk in the 2012 working age population (LR_p_)	0.3 %	0.1 %
Total number of mesotheliomas in the 2012 population until 2094 (D_p_)	23,819	10,679
Lifetime excess risk of mesothelioma for those exposed to asbestos in 2012 (LR_x_)	4.26 %	3.34 %
Future number of mesotheliomas in the working age population due to occupational asbestos exposure in 2012 (FEN)	7549	22
Fraction of future mesotheliomas in the working age population due to occupational exposure in 2012 (FEF)	0.317	0.002

Using the AF approach we estimated that there would be 240 mesotheliomas in men occurring in 2052 due to exposure in 2012 with an attributable fraction of 39 %. In women there would be 1 mesothelioma and the AF would be <1 %.

We undertook sensitivity testing of the models in relation to the outputs in Table 1 for five primary inputs (numbers exposed, future population, future incident cases, future mortality rates, and RR).

In both sexes, when we varied the number of people exposed to asbestos (but not the future incident cases), a flow on effect resulted in a sensitive inverse relationship in the calculation of *LR*_*x*_ and subsequently a significant sensitivity change in *FEN* and *FEF*. These changes were more prominent in males, as females have a comparatively lower number exposed.

When we changed the baseline population numbers used in the lifetime risk model while keeping the number of exposed the same, we saw an inverse relationship between population numbers and *LR*_*x*_*, FEN,* and *FEF.*

Bender’s model [7] may be more sensitive to incidence than to mortality and sensitivity analysis confirmed this. Altering the mortality rates used in the double decrement tables affected PYAR and resulted in small inverse changes in *LR*_*p*_*, LR*_*x*_*,* and *FEN* (which were less than proportional). However if the incidence rates of future cancers were altered the changes in the same three outcomes were proportional.

Changes in *RR* resulted in a significant change in *LR*_*x*_, *FEN* and *FEF* for both sexes. Changes were larger in females due to the comparative increase in the calculation of *LR*_*x*_.

## Discussion

We have presented a novel approach to calculate the future effect of exposures occurring now. This method can be used for a range of different exposures and diseases for which exposure data are available for an index year and there are appropriate data for predicted future rates of disease. We have written an R program which is available on request for other researchers wishing to use this method.

### Comparisons with other methods

The FEF as calculated with this method is not directly comparable to the attributable risk percent as calculated with the attributable risk approach. As shown in Fig. 1, the approaches are estimating different concepts and are using different data. For example, occupational use of asbestos in Australia was much more widespread in the past than it was in 2012 and people who are still working in 2012 may have previously been occupationally exposed to asbestos. It might be more appropriate for the AF method to use the proportion of workers in 2012 who have ever been exposed to asbestos at work rather than a point prevalence of exposure in 2012. However such data are unavailable.

Only one other study has calculated the future burden of mesothelioma due to occupational exposure to asbestos - Hutchings et al. estimated that, by 2060, about 73 % of mesotheliomas will still be attributable to exposure to asbestos that is occurring at work beteween now and then [15]. The major difference between this method and the FEF method is that we do not include future exposure to asbestos.

Several previous studies have estimated the proportion of current mesotheliomas due to past occupational exposure to asbestos. A UK study used actual mesothelioma counts and concluded that 97 % of male mesothelioma deaths and 82 % of female mesothelioma deaths in 2004 were due to past occupational or environmental exposure to asbestos [16]. In the Australian Mesothelioma Registry in 2014, 74 % of mesotheliomas in males and 4 % in females had an occupational cause [17]. A French study examined work histories of mesothelioma cases and controls and calculated AFs of 83 % for men and 42 % for women [18]. Other investigators have assumed that all mesotheliomas are due to occupational exposure, that is, that the AF is 100 % [19]. In Australia, the number of people exposed to asbestos, particularly at high levels, has fallen markedly in the previous 50 years, and more exposure is occurring in non-occupational scenarios [20] so these proportions will decrease in the future.

### Assumptions and implications of the assumptions

A major consideration in the use of the FEF method is the definition of the exposed population. In our example we used the prevalence of exposure in the index year. This is similar to the usual way of implementing the attributable fraction approach when a point prevalence of exposure in one historical year is used [3]. An alternative is to use the prevalence of “ever exposed” in the index year or historical year, if these data were available. Doing this would take account of the effect of duration of exposure which contributes to the estimates of risk (RR) used. The difference between point prevalence and prevalence of “ever exposed” would be likely to be greater for exposures which change over time. For example, occupational exposures change when a worker changes a job, and therefore the prevalence of “ever exposed” is likely to be higher than the prevalence of exposure at a single point in time and use of the point prevalence will underestimate the number of future cases. However, this may not be the case for relatively constant exposures such as obesity and alcohol intake which are less likely to change markedly over time. A method of adjusting point prevalences with turnover rates to more closely resemble the “ever exposed” prevalence has also been used [21].

When using the FEF method it is necessary that the chosen RR matches the definition of exposure used for the estimation of exposure prevalence. In our example, the exposure information was limited to whether there was or was not exposure to asbestos in the index year and we had no information on how long exposure had occurred nor the intensity of that exposure although a level of intensity may be available for other agents. We used a point prevalence estimate of exposure in the index year and implicitly assumed that there was a normal distribution curve with regard to the length of exposure and the level of exposure. There would be people exposed in the index year who had very low exposure for a short period as well as people who had high exposure for a long period. We used a RR from the general working population which would also have included people with a range of durations and levels of exposure. Although this RR is related to “ever exposed” it is appropriate to apply to the number of people exposed only in the index year, rather than also including those ‘ever exposed’ prior to but not in that year. Because the effect measure has a significant impact on the results it is important that the most appropriate and best justified measure is chosen. Meta-analyses and pooled analyses may be the best source for the effect measure if these are available.

We did not include a latency period in our estimates of lifetime risk. Similarly to the assumption of previous exposure, we assumed that there was no need for a latent period as some people exposed in the index year had been exposed for a long time and may have developed mesothelioma soon after the index year. An advantage of the LR approach is that it avoids the need to account for latency, as it estimates disease appearing over a lifetime rather than in a specific year, so that timing is not relevant.

Life tables were used for estimating the projected future person-years at risk. While mortality rates in the future may change, sensitivity analysis found that the FEN and FEF in the exposed were sensitive to changes in prevalence of exposure, population numbers, and relative risks, but much less sensitive to changes in mortality.

A limitation of the FEF method is the accuracy of projections of future disease. The validity of a projection will vary by the disease outcome being modelled. In our example, high quality national cancer registry data were available for the previous 30 years, but this is not always the case for other diseases or other countries. The future number of cases depends on the change in size and age structure of the population (which is relatively easy to predict), as well as changes in the rates of disease (which are more difficult to predict) [22]. We used CanProj which estimates likely future trends based on what has been observed in the past and does not take into account possible changes in risk factors (such as changes in smoking patterns). We projected rates for over 80 years into the future, so errors are likely to be large, others may limit the projection to a shorter interval. Even so, cancer is one of the diseases for which there is a relatively good range of options for projecting future rates. Most of the approaches are based on age-period-cohort models including a recently produced Bayesian method [23]. An alternative to using a forward projection model that takes account of competing risks causing the same disease outcomes (step 5 in the example) would be to apply a constant disease rate (for t = 0, e.g., 2012) to projected PYAR.

### Policy applications

In the FEF method, exposure can easily be varied to predict the effect of various control measures. For example, in the case of cigarette smoking it is possible to model the effect of policies such as reducing tar content of cigarettes, mandating plain packaging, or increasing cost. Because the estimations are based on changes to current exposure, they are readily understood by policy makers. In addition, the exposures are current, so are of more salience to policy makers than exposures occurring in the past (e.g., it may be easy to disregard mesotheliomas arising from past exposure to asbestos in the belief that current exposure is negligible).

### Optional extensions to the FEF model

Researchers with more detailed exposure data may be able to match their exposure categories with several RRs. For example, if there are population data on the prevalence of exposure to cigarette smoking for the index year, it may be possible to use different RRs for the different categories of pack-years. This would allow prediction of the effect of reducing exposures (e.g., smoking fewer cigarettes a day) rather than completely eliminating exposures.

Multiple exposures or outcomes can be accounted for using the product of complements method [2]: In this method the combined FEF for one outcome is equal to the complement of the product of the complement of each FEF for k exposures.

$$ FE{F}_{combined}=1 - {\displaystyle {\prod}_k\left(1-FE{F}_k\right)} $$

The combined FEF can be multiplied by the number of expected outcomes in the population to obtain the combined FEN for one outcome and multiple exposures. Combined FENs for different outcomes can be summed to obtain the overall number of attributable outcomes.

In conclusion, the future excess fraction method offers a different way of expressing the burden of disease which is readily understandable by the general public and by those with the ability to influence public health decisions.

### Ethics

This manuscript describes a new method, and used publically available data sets for the example therefore no ethics approval was required.

### Consent

No individual or identifiable data were used in this manuscript and therefore no consent was required.

### Availability of data and materials

All data sources are referenced.

### Trial registration

This manuscript does not report the results of a controlled health-care intervention and is not a trial.

## Appendix

The formula for calculating lifetime excess risk (*LR*_*x*_) for an individual exposed to the agent in the index year is:

1 $$ L{R}_x=L{R}_u*\left(RR-1\right) $$

where (*RR - 1*) is the excess risk in the exposed relative to the unexposed and *LR*_*u*_ is the baseline risk in the population in the absence of exposure. Since *LR*_*u*_ is not usually available we have derived a version of this formula which does not require an estimate of *LR*_*u*_.

The lifetime risk in the population in a specific year is equal to the total number of estimated disease cases (*D*_*it*_) divided by the total number in the population in the index year (*N*_*p(t = 0)*_).

2 $$ L{R}_p = {\displaystyle {\sum}_{i=l}^{i=u}\frac{{\displaystyle {\sum}_{j=l}^{j=u}}{D}_{ij}}{N_{p\left(t=0\right)}}} $$

In this formula, the total number of disease cases is equal to those occurring in the unexposed (*FN*_*u*_), plus those occurring in the exposed which would occur without exposure (baseline incident cases = *FN*_*eb*_) plus those occurring in the exposed due to the exposure (excess cases = FEN).

3 $$ L{R}_p=\frac{F{N}_u+F{N}_{eb}+ FEN}{N_{p\left(t=0\right)}} $$

4 $$ L{R}_p = \frac{\left(L{R}_u*{N}_{u\left(t=0\right)}\right)+\left(L{R}_u*{N}_{e\left(t=0\right)}\right)+\left(L{R}_x*{N}_{e\left(t=0\right)}\right)}{N_{p\left(t=0\right)}} $$

Solving for *LR*_*u*_ and substituting N_p(t = 0)_ for (N_u(t = 0) *+*_ N_e(t = 0)_)

5 $$ \begin{array}{l}L{R}_p*{N}_{p\left(t=0\right)}=L{R}_u*\left({N}_{u\left(t=0\right)}+{N}_{e\left(t=0\right)}\right)+\left(L{R}_x*{N}_{e\left(t=0\right)}\right)\hfill \\ {}\left(L{R}_P*{N}_{p\left(t=0\right)}\right)-\left(L{R}_x*{N}_{e\left(t=0\right)}\right)=L{R}_u*\left({N}_{u\left(t=0\right)}+{N}_{e\left(t=0\right)}\right)\hfill \\ {}\left(L{R}_P*{N}_{p\left(t=0\right)}\right)-\left(L{R}_x*{N}_{e\left(t=0\right)}\right)=L{R}_u*{N}_{p\left(t=0\right)}\hfill \\ {}L{R}_u=\frac{\left(L{R}_P*{N}_{p\left(t=0\right)}\right)-\left(L{R}_x*{N}_{e\left(t=0\right)}\right)}{N_{p\left(t=0\right)}}\hfill \end{array} $$

Substituting this in Eq. (1) and simplifying:

6 $$ {\mathrm{LR}}_x=\frac{\left[\left({\mathrm{LR}}_p*{\mathrm{N}}_{p\left(t=0\right)}\right)-\left({\mathrm{LR}}_x*{\mathrm{N}}_{e\left(t=0\right)}\right)\right]*\left(\mathrm{R}\mathrm{R}-1\right)}{{\mathrm{N}}_{p\left(t=0\right)}} $$

7 $$ \begin{array}{l}L{R}_x*{\mathrm{N}}_{p\left(t=0\right)}=\left(L{R}_P*{\mathrm{N}}_{p\left(t=0\right)}\right)\left(RR-1\right)-\left(L{R}_x*{\mathrm{N}}_{e\left(t=0\right)}\right)\left(RR-1\right)\hfill \\ {}\left(L{R}_x*{\mathrm{N}}_{p\left(t=0\right)}\right)+\left(L{R}_x*{\mathrm{N}}_{e\left(t=0\right)}\right)\left(RR-1\right)\kern0.5em  = \left(L{R}_P*{\mathrm{N}}_{p\left(t=0\right)}\right)\left(RR-1\right)\hfill \\ {}L{R}_x\left({\mathrm{N}}_{p\left(t=0\right)}+{\mathrm{N}}_{e\left(t=0\right)}\left(RR-1\right)\right) = \left(L{R}_P*{\mathrm{N}}_{p\left(t=0\right)}\right)\left(RR-1\right)\hfill \\ {}\ L{R}_x=\frac{\left(L{R}_P*{\mathrm{N}}_{p\left(t=0\right)}\right)\left(RR-1\right)}{{\mathrm{N}}_{p\left(t=0\right)}+{\mathrm{N}}_{e\left(t=0\right)}\left(RR-1\right)}\hfill \\ {}L{R}_x=\frac{\left(L{R}_P*{\mathrm{N}}_{p\left(t=0\right)}\right)\left(RR-1\right)}{\left({\mathrm{N}}_{u\left(t=0\right)}+{\mathrm{N}}_{e\left(t=0\right)}\right)+{N}_e\left(RR-1\right)}\hfill \\ {}{\mathrm{LR}}_x=\frac{\left({\mathrm{LR}}_P*{\mathrm{N}}_{p\left(t=0\right)}\right)\left(\mathrm{R}\mathrm{R}-1\right)}{{\mathrm{N}}_{u\left(t=0\right)}+{\mathrm{N}}_{e\left(t=0\right)}*\mathrm{R}\mathrm{R}}\hfill \end{array} $$
